# Data-Efficient Unsupervised Recalibration of Calorimeter Sensor Arrays Using Wasserstein Adversarial Learning

**DOI:** 10.3390/s26165024

**Published:** 2026-08-07

**Authors:** Saraa Ali, Vladimir Bocharnikov, Fedor Ratnikov, Mikhail Hushchyn, Artem Ryzhikov, Denis Derkach

**Affiliations:** Laboratory of Methods for Big Data Analysis, HSE University, Pokrovsky Boulevard 11, Moscow 109028, Russia; vbocharnikov@hse.ru (V.B.); fratnikov@hse.ru (F.R.); mhushchyn@hse.ru (M.H.); aryzhikov@hse.ru (A.R.); dderkach@hse.ru (D.D.)

**Keywords:** sensor-array calibration, sensor drift, sensor aging, AI-enabled sensor monitoring, calorimeter sensors, Wasserstein distance, WGAN, unsupervised recalibration

## Abstract

Large distributed sensor arrays require repeated recalibration as radiation damage, material aging, gain variation, and readout drift alter channel responses. We studied a high-granularity calorimeter as a large sensor array and addressed unsupervised recalibration from two unpaired datasets: a nominal reference response and an aged response with attenuated cell-wise signals. Aging was modeled by a deterministic sensor-wise base field with reading-level stochastic variation; the base coefficients were used only for post-training evaluation. We evaluated a Wasserstein adversarial calibration field against an evaluation-only global-mean coefficient predictor and two non-adversarial estimators, a per-cell mean-energy ratio and independent per-cell Wasserstein matching. The adversarial objective supplied an adaptive event-level discrepancy over the full sensor array. In a hierarchical data-efficiency study with four reference/aged sampling-seed combinations and three configured-seed adversarial fits per seed combination, the adversarial method achieved an RMSE from 0.0238±0.0014 to 0.0173±0.0012 and a positive $R^{2}$ from 0.828±0.022 to 0.910±0.013 across the tested event counts. Its RMSE was also below the approximately 0.0579 global-mean reference at every event count, demonstrating the recovery of cell-wise coefficient variation beyond the global mean. The mean-energy-ratio and Wasserstein-only estimators remained below the $R^{2}=0$ reference in this sparse benchmark.

## 1. Introduction

Large sensor arrays are being increasingly used in scientific instrumentation, industrial monitoring, medical imaging, and particle-detection systems. Their performance depends not only on the quality of individual sensors, but also on the stability of thousands of coupled channels over long operating periods. In practice, sensor responses are not perfectly stationary. Radiation exposure, material aging, photodetector gain changes, electronics drift, and environmental effects can alter the response of individual channels, shifting both local signal distributions and system-level observables [1,2,3].

High-granularity calorimeters [4] are a demanding example of this sensor-array problem. They measure particle energies by recording spatially distributed energy deposits in many readout cells, so long-term stability is essential for reliable reconstruction and monitoring. Conventional calorimeter recalibration is detector-specific and combines several layers: channel equalization and response constants established with laboratory, commissioning, or test-beam measurements; the hardware, optical, or source-based monitoring of gain changes; and in situ physics constraints that update relative channel responses and absolute energy scales during operation [1,2,3]. The method studied in this paper is intended as a complementary data-driven update between validated detector states, particularly when frequent sensor-level labels are unavailable. This advancement is achieved using Wasserstein-distance distribution matching. With AI-enabled monitoring, such updates can be supported using routinely collected detector data [5,6].

We consider the practical setting in which a reference dataset from a nominal sensor-array state and a second dataset from an aged state are available. For deployment, feasibility is not only a question of whether the aging coefficients can be recovered eventually. It is also a question of data efficiency: how does the recovery accuracy change as the available event sample increases? This is a statistical issue because a high-granularity calorimeter contains many sensor-level aging coefficients, while each event provides a sparse, structured shower pattern rather than direct observations of every coefficient. We therefore characterized the accuracy over four tested event counts and compared an independent per-cell optimal-transport baseline with an adversarial method that uses the full sensor-array response; we did not interpret this finite sweep as establishing a universal minimum event requirement.

Earlier studies introduced the WGAN-inspired idea for calorimeter response correction and demonstrated its feasibility in fixed data regimes [7,8], but no studies about data efficiency have been provided.

We formulated aging-aware sensor-array recalibration as an unsupervised distribution-matching problem. The unknown physical parameters are cell-wise multiplicative aging coefficients. Rather than predicting labels from examples, the method adjusts the estimated coefficients until the corrected aged response distribution matches the reference distribution. This perspective connects sensor drift correction and detector calibration to dataset shift, simulation-based parameter inference, domain alignment, optimal transport, and Wasserstein adversarial learning [9,10,11,12,13].

The contribution of this article is a systematic framework for evaluating the approach’s reliability and practical limitations. The study combines hierarchical replication across independent data samples and configured seeds, an expanded four-method benchmark, evaluation against a global-mean reference, and controlled characterization across four event counts. Coefficient-level metrics are complemented by local cell-response and event-level validation, while identifiability is treated explicitly as a limitation of distribution-matching calibration. Together, these elements extend the earlier proof-of-concept studies into a comparative assessment of empirical recoverability, data efficiency, and robustness.

## 2. Materials and Methods

### 2.1. Sensor-Array Calibration Problem

Let $X\in R_{+}^{L\times H\times W}$ denote a calorimeter event represented as a tensor of cell energies, with cell index i=(ℓ,u,v). We assume access to two unpaired datasets:

(1) $$\begin{array}{cc} D_{\mathrm{ref}} & ={\left\{X_{\mathrm{ref}}^{\left(n\right)}\right\}}_{n=1}^{N_{\mathrm{ref}}}, \end{array}$$

(2) $$\begin{array}{cc} D_{\mathrm{aged}} & ={\left\{X_{\mathrm{aged}}^{\left(m\right)}\right\}}_{m=1}^{N_{\mathrm{aged}}}. \end{array}$$

Throughout the paper, *reference* and *undamaged* denote the nominal detector response, while *aged* denotes the attenuated detector response; figures may use the equivalent term *damaged* for this same aged sample. Each cell is treated as a sensor channel whose response may drift independently, so calibration is a large-scale parameter-estimation problem rather than a single global energy-scale correction.

**Figure 1:**
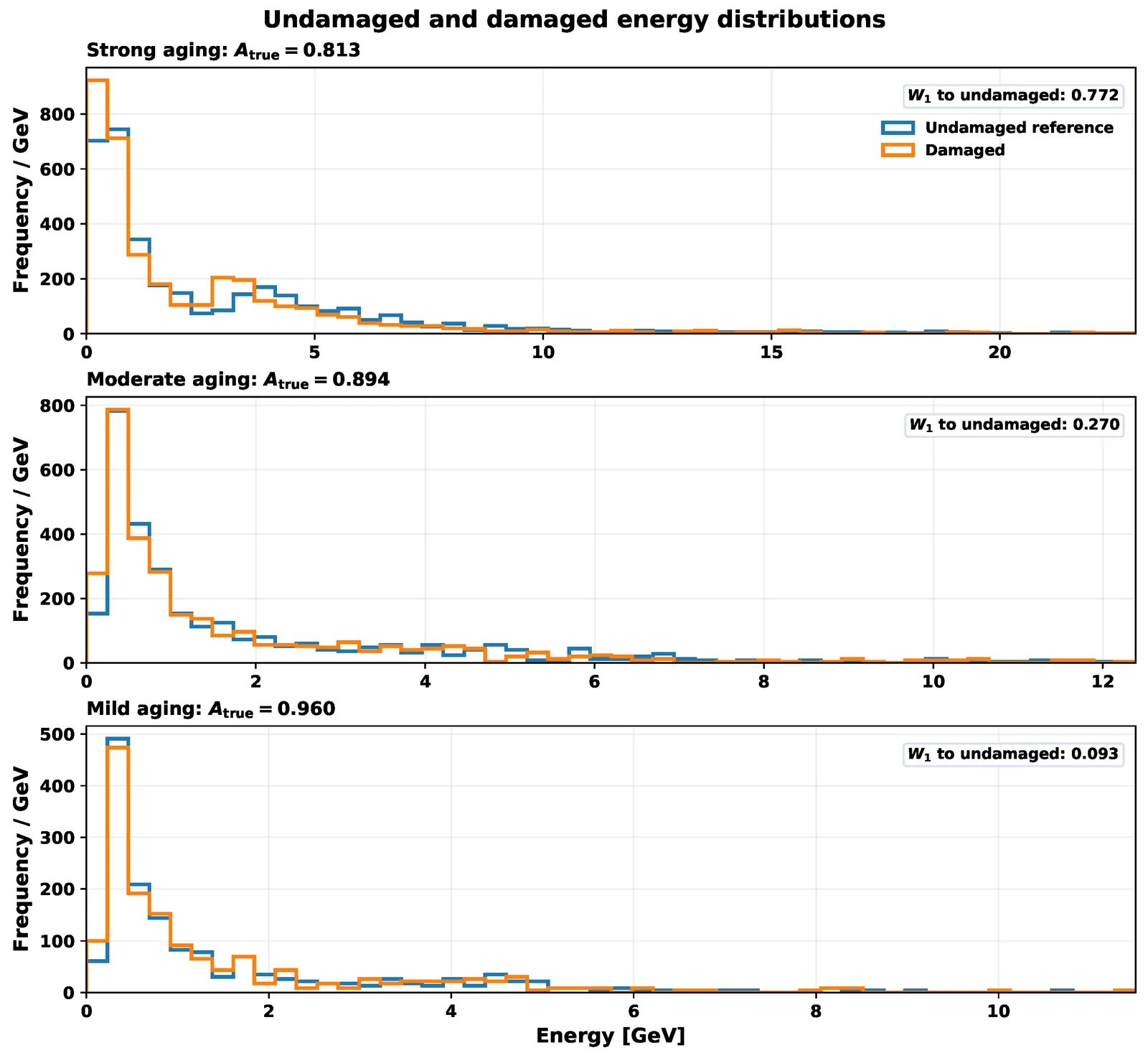
Representative pre-calibration energy-reading distributions for three calorimeter cells. Reference (undamaged) distributions are compared with aged responses after injected attenuation, illustrating the cell-wise shifts that the unsupervised calibration must infer from unpaired samples.

In simulations, the aged detector response is generated by applying a fixed cell-wise coefficient field to nominal calorimeter events:

(3) $$X_{\mathrm{aged}}^{\left(i\right)}=A_{i}X_{\mathrm{ref}}^{\left(i\right)},\;A_{i}\in \left(0,1\right],$$

where $A_{i}$ is the unknown aging coefficient, or aging factor, for cell *i*. During training, however, the reference and aged samples are treated as unpaired datasets. In the experiments considered here, the stochastic coefficient perturbation has the scale $1.00\times 10^{-2}$. Values close to one correspond to nearly unaffected sensors, while smaller values indicate stronger attenuation. For an individual cell, aging shifts the empirical energy-reading distribution relative to its nominal response; across the detector, these shifts combine into a structured event-level distribution change. The calibration task is to estimate ${\hat{A}}_{i}$ or, equivalently, the inverse correction $1/{\hat{A}}_{i}$, using only samples from $D_{\mathrm{ref}}$ and $D_{\mathrm{aged}}$ during optimization. The injected $A_{i}$ values are held out for evaluation through the RMSE, $R^{2}$, and aging-coefficient scatter plots. Figure 1 illustrates the effect of aging at the level of individual sensors by showing example energy-reading distributions for three calorimeter cells with different values of $A_{i}$. The undamaged distribution was taken from the reference sample, while the aged distribution was obtained after applying the injected aging coefficient. As expected, the discrepancy between the reference and aged distributions was larger for more strongly attenuated cells.

### 2.2. Calibration Methods

#### 2.2.1. Wasserstein-Only Cell Search

The most direct distribution-matching baseline treats each cell independently. We refer to this method as the Wasserstein-only (WS-only) baseline. For cell *i*, let $P_{i}$ be the empirical distribution of reference energies and $Q_{i}$ be the empirical distribution of aged energies. We define $C=\left\{c_{1},\ldots ,c_{K}\right\}\subset \left[c_{\min},c_{\max}\right]$ as the finite grid of candidate aging coefficients. In the reported experiments, C contains 10,000 evenly spaced values over [0.5,1.0]. We estimate

(4) $${\hat{A}}_{i}^{\mathrm{WS}}=\arg\min_{c\in C}W_{1}\left(P_{i},\;Q_{i}/c\right),$$

where $W_{1}$ is the one-dimensional Wasserstein distance and the superscript WS denotes the WS-only estimate. This baseline is appealing because it is interpretable, requires no learned critic, and can use unequal numbers of reference and aged samples. For unweighted one-dimensional empirical distributions, the computation can be accelerated by sorting each cell’s samples once and evaluating the candidate grid through quantile intervals. The limitation is that Equation (4) discards joint shower information. A calorimeter response is not merely a collection of independent cell histograms; cell occupancies, layer profiles, local shower shapes, and correlations across neighboring channels provide information about which calibration fields are plausible.

#### 2.2.2. Evaluation Controls and Mean-Energy-Ratio Baseline

We added two test methods. The first is an evaluation-only global-mean coefficient predictor that assigns the true mean aging coefficient over the evaluated cells to every cell. This predictor uses ground-truth information unavailable during unsupervised training or deployment, and is therefore not a calibration method. It is included only as an evaluation reference: under the coefficient-of-determination definition used here, it has $R^{2}=0$, and its RMSE is the standard deviation of the true cell-wise coefficient field.

The second control is a deployable, non-adversarial per-cell mean-energy-ratio estimator. For each active cell, we divide its mean aged energy by its mean reference energy. Both means use the full number of events as the denominator, so events with no retained reading in that cell contribute zero rather than being discarded. The resulting ratio is constrained to the same physical interval [0.5,1.0] used by the other estimators. This baseline is simple and interpretable, it estimates each cell independently and it can confound response attenuation with finite-sample differences in the occupancy and deposited-energy composition between the two unpaired samples.

#### 2.2.3. Adversarial Wasserstein Calibration

Following [14], we therefore used a WGAN-style objective, but reinterpreted the generator. In a standard GAN, the generator maps random noise to synthetic samples [15,16,17]. Here, the generator is a deterministic calibration module parameterized directly by cell-wise aging coefficients:

(5) $$\hat{A}={\left\{{\hat{A}}_{i}\right\}}_{i=1}^{LHW}.$$

For applying a learned calibration to aged events, the inverse correction transform is

(6) $$T_{\hat{A}}{\left(X_{\mathrm{aged}}\right)}^{\left(i\right)}=\frac{X_{\mathrm{aged}}^{\left(i\right)}}{{\hat{A}}_{i}+\delta },$$

with a small δ for numerical stability. Equivalently, one may train in the forward direction by applying ${\hat{A}}_{i}$ to reference events and matching the aged distribution. In both cases, the learned field $\hat{A}$ is the interpretable output.

Let $C_{\phi }$ be a CNN critic that maps a calorimeter tensor to a scalar score. The critic is trained to distinguish reference events from corrected aged events through the WGAN objective:

(7) $$\max_{\phi \in \Phi }E_{X∼p_{\mathrm{ref}}}\left[C_{\phi }\left(X\right)\right]-E_{Y∼p_{\mathrm{aged}}}\left[C_{\phi }\left(T_{\hat{A}}\left(Y\right)\right)\right],$$

subject to an approximate 1- Lipschitz constraint. The calibration field is optimized in the opposite direction:

(8) $$\min_{\hat{A}}-E_{Y∼p_{\mathrm{aged}}}\left[C_{\phi }\left(T_{\hat{A}}\left(Y\right)\right)\right].$$

**Figure 2:**
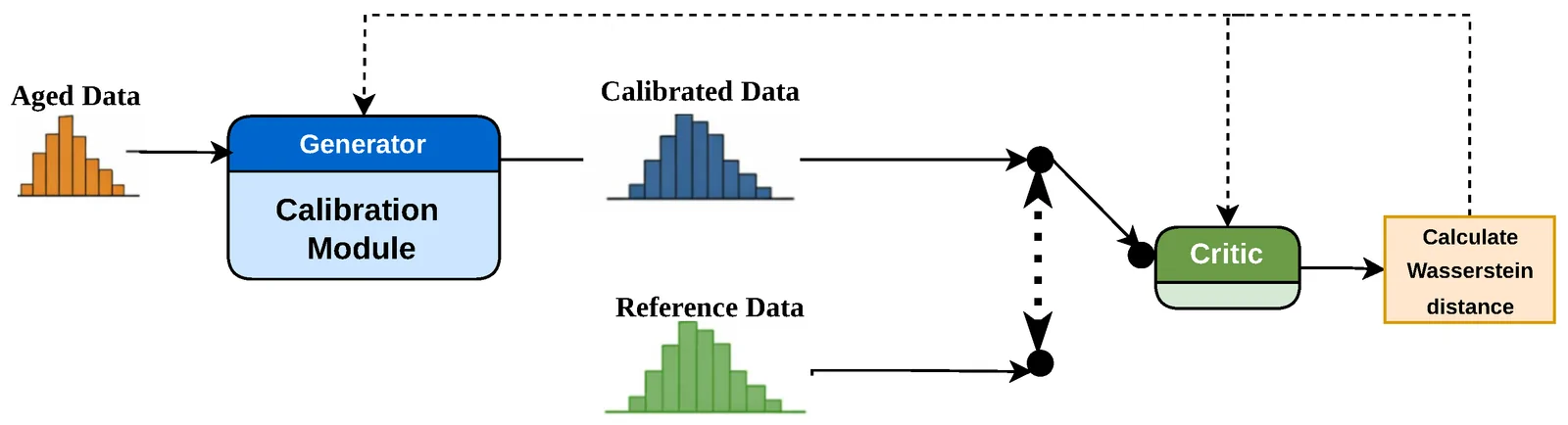
Wasserstein adversarial calibration architecture. The calibration module is a deterministic field of aging coefficients, not a generator that samples from latent noise. A learned critic compares structured calorimeter events and supplies the WGAN-style Wasserstein discrepancy used to update the calibration field.

In our implementation, the critic treats calorimeter layers as channels and uses two-dimensional convolutions over the transverse grid. This architecture is important because the adversarial signal is not fixed to a preselected one-dimensional histogram; it adapts to event-level discrepancies in the shower structure, layer profiles, and correlated cell activity. Training procedure: The calibration workflow begins with two unpaired samples: a nominal reference response and an aged response. For each sensor cell, a trainable calibration value is initialized. During training, the current calibration values are applied to aged events, producing a corrected aged response that is compared with the reference response. The critic is then updated to identify event-level differences that still distinguish the two samples, while the calibration values are updated to reduce these differences. After every update, the calibration values are constrained to remain within the physically allowed aging range. The final result is a learned sensor-wise calibration field, which can be used as an inverse correction for aged detector data. Figure 2 summarizes this training workflow.

### 2.3. Experimental Setup

We evaluated on Monte Carlo calorimeter data produced with the Geant4 framework [18]. The underlying simulation contained single $\pi^{-}$ hadronic shower events with a kinetic energy of 10 GeV. The detector geometry followed a high-granularity sampling-calorimeter configuration representative of next-generation collider instrumentation [19]. It consisted of longitudinal absorber layers made of 20 mm copper plates, interleaved with active polystyrene scintillator tiles. Each active layer was segmented into a 24×24 matrix of 5 mm thick tiles, resulting in the fine spatial sampling of the deposited-energy pattern. In the prepared learning dataset, each event was embedded in a tensor representation, with the layer index used as the channel dimension and the transverse tile coordinates used as spatial dimensions. The reported aging-coefficient metrics were computed only on the active cell coordinates shared by the reference and aged samples, while the remaining tensor entries retained the fixed detector grid required by the convolutional model. The reference sample represented the corrected nominal detector response. To model a later aged detector state, we applied synthetic degradation to the same sensor-array geometry and treated the resulting shifted response distribution as an unpaired aged sample.

In this simulation study, the reference data were the nominal Geant4 events and therefore did not come from a particular deployed calorimeter. In an operational application, a reference sample would need to be collected for the calorimeter, or for a detector module demonstrated to have an equivalent response, during commissioning after conventional calibration or during a later period with an independently validated stable response. Reference and monitoring samples would require the same geometry, readout definition, and preprocessing as well as a controlled event population. The learned cell-wise field is consequently detector- and configuration-specific; it is not assumed to transfer unchanged to a different calorimeter. A separate fit or validated update would be required for each detector configuration, and the present method is not directly applicable when no trustworthy nominal reference state is available.

The degradation is modeled by assigning each readout channel a cell-specific aging coefficient $A_{i}\in \left(0,1\right]$. In the synthetic aging model, $A_{i}$ has a linear dependence on the integrated energy deposition in cell *i* over the entire simulated dataset, and cells with a greater accumulated deposited energy receive lower response coefficients. This construction mimics the radiation-induced loss of sensitivity under prolonged exposure and is used as a controlled proxy for detector aging rather than as a detector-specific material damage law. A small stochastic perturbation of the scale $1.00\times 10^{-2}$ models finite channel-to-channel variation. The aged response is produced by multiplying each cell signal by its coefficient, so strongly exposed cells show a larger shift away from the nominal response. The task is then to infer these hidden sensor-wise coefficients from the distributional shift between the nominal reference data and the aged data, without using event-by-event correspondence or coefficient labels during optimization. During training, the WGAN model sees only reference and aged events; true aging coefficients are used after training to compute aging-coefficient metrics.

The data-efficiency study used disjoint reference and aged event samples with 50,000, 90,000, 150,000, and 250,000 events per detector state. All event-count settings were generated from the same underlying detector geometry, simulation sample, and aging-generation rule, so the detector configuration and aging scenario remained fixed throughout the sweep. The primary independent units were four disjoint reference/aged sampling-seed combinations: (0,1), (2,3), (4,5), and (6,7). For every event count and seed combination, the adversarial method was fitted with configured seeds 42, 43, and 44, giving 12 WGAN fits per event count. In the main comparison, we first averaged the three WGAN metrics within each seed combination and then reported the mean and sample standard deviation across the four seed-combination averages. The individual fits are reported in Supplementary Table S1. Because the configured seed also controls the stochastic synthetic-aging perturbation in the current preparation pipeline, these repetitions measure the robustness to the combined configured-seed variation rather than an isolated optimizer-initialization effect. The finite event-count sweep characterizes the performance at the tested sample sizes, but does not identify a universal number of events required for deployment.

The WGAN calibration field $\hat{A}$ is represented by one trainable tensor of shape 40×24×24 and is initialized to unity, ${\hat{A}}_{i}=1$ for all cells. The critic is a five-convolution two-dimensional CNN that treats the 40 calorimeter layers as input channels over the 24×24 transverse grid. Its channel sequence is 40→64→128→128→64→1: the first four convolutions use 3×3 kernels, padding one, and LeakyReLU activations with slope 0.2; the three hidden blocks include affine instance normalization; and the final convolution uses a 4×4 kernel followed by 21×21 max pooling to produce one scalar critic score per event. The convolutional weights were initialized with Xavier-normal initialization. Both the critic and the calibration field were optimized with Adam using the learning rate $2.00\times 10^{-4}$ and betas (0.500,0.999), with a batch size of 8192. We used one critic update per calibration-field update, clipped critic weights to $\left[-1.00\times 10^{-2},1.00\times 10^{-2}\right]$, and clamped $\hat{A}$ to [0,1] after each update. The WS-only baseline searched 10,000 candidate aging coefficients in [0.5,1.0] for each cell. All four benchmark entries used the same active-cell definition and the injected aging coefficients only as post-training evaluation targets.

## 3. Results

### 3.1. Aging-Coefficient Recovery

**Figure 3:**
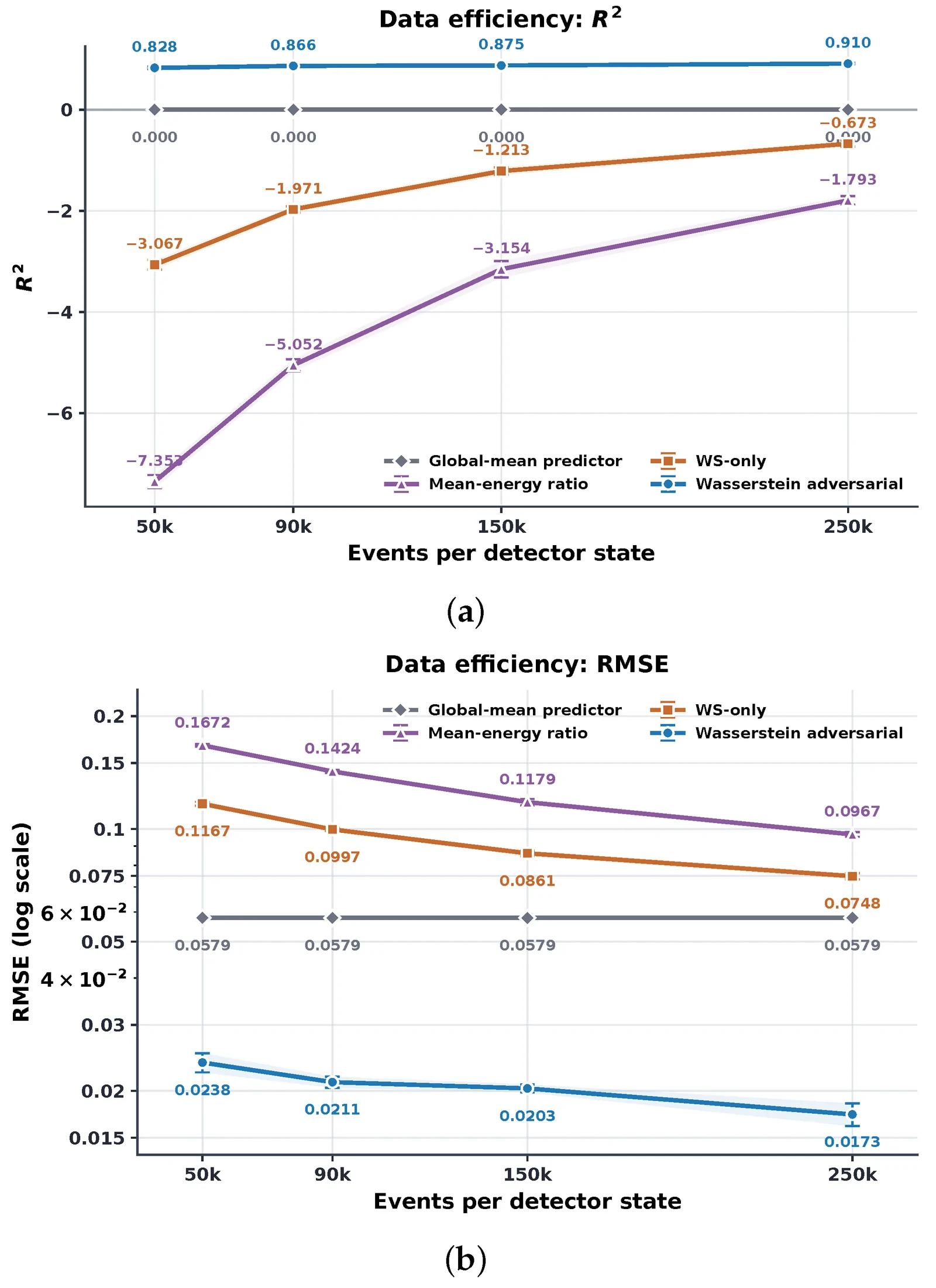
Data-efficiency benchmark for cell-wise aging-coefficient recovery: (**a**) coefficient of determination, $R^{2}$, and (**b**) RMSE on a logarithmic scale. The global-mean predictor assigns the true mean coefficient to every active cell and defines the evaluation reference $R^{2}=0$. All methods were evaluated on the same active cells. The numerical values underlying this figure are reported in Table 1.

Figure 3 shows the data-efficiency comparison benchmark for all four methods. The global-mean predictor provided an evaluation reference with $R^{2}=0$ and a constant RMSE of 0.0579. The Wasserstein adversarial method is the only evaluated method that achieved a positive $R^{2}$ and an RMSE below the global-mean reference at every tested event count. With 50,000 events per detector state, the adversarial method reached an RMSE of 0.0238±0.0014 and $R^{2}=0.828\pm 0.022$; with 250,000 events per state, it reached an RMSE of 0.0173±0.0012 and $R^{2}=0.910\pm 0.013$. For the adversarial method, each point first averaged the three configured-seed fits within each seed combination; the displayed mean and error bar were then calculated across the four seed-combination averages. The mean-energy-ratio and WS-only estimators improved as the event count increased, but remained below the $R^{2}=0$ reference throughout the tested range.

Table 1 reports the comparison with the global-mean, per-cell mean-energy-ratio controls, and WS-only values. The adversarial method had an RMSE below the global-mean predictor and a positive $R^{2}$ at every event count, indicating the recovery of cell-wise coefficient variation beyond the global mean. The mean-energy-ratio and WS-only estimators both remained below the $R^{2}=0$ reference, indicating that these independent per-cell estimators were insufficient for the present sparse, unpaired benchmark. Increasing the event count improved the methods, but the independent one-dimensional baselines remained dominated by finite-sample and marginal-identifiability limitations. The adversarial critic instead evaluated structured full-event responses, allowing correlations between layers, neighboring cells, and shower shapes to constrain the same calibration field.

Supplementary Table S1 reports all 12 adversarial fits at each event count. It makes the within-combination configured-seed variation visible without treating those fits as 12 independent data samples. The main comparison therefore avoids pseudo replication by retaining the four independent sampling-seed combinations as its statistical units. As noted in the experimental setup, this table demonstrates the robustness to the pipeline’s configured-seed variation.

To examine whether coefficient recovery depends on how frequently a cell is observed, we counted the number of nonzero readings for each active cell in the complete nominal dataset. The cells were ranked by this count and divided into three approximately equal-sized groups representing low, medium, and high occupancy. Group membership was defined once and then kept fixed for all event counts, seed combinations, and configured seeds. Supplementary Table S2 reports the corresponding RMSE values at (50,000) and (250,000) events per detector state. The high-occupancy group had the lowest adversarial RMSE at both event counts, indicating that frequently observed cells are better constrained. Low- and medium-occupancy cells were recovered less accurately, consistent with the identifiability limitation discussed below. This occupancy analysis indicates where coefficient recovery is more reliable, but it does not establish the uniqueness of the inferred calibration field.

The $R^{2}$ values were interpreted in the standard coefficient-of-determination sense. We computed $R^{2}=1-\sum_{i}{\left({\hat{A}}_{i}-A_{i}\right)}^{2}/\sum_{i}{\left(A_{i}-\bar{A}\right)}^{2}$; thus, a negative $R^{2}$ means that a predictor has a larger squared error than the global-mean predictor [20]. The negative values for both independent estimators therefore preclude using their relative gap to establish a strong methodological advantage. The more informative observation is that the adversarial method was consistently above zero and had a lower RMSE than the global-mean reference. This result supports the hypothesis that a full-event shower structure provides information about local coefficient variation that the tested cell-wise marginal estimators do not recover. Across the tested event-count range, the adversarial mean RMSE decreased from 0.0238 to 0.0173, while the mean $R^{2}$ increased from 0.828 to 0.910. These observations characterize the evaluated sample budgets; selecting an operational event requirement would additionally require detector-specific precision targets and validation on independent monitoring periods.

### 3.2. Qualitative Agreement

**Figure 4:**
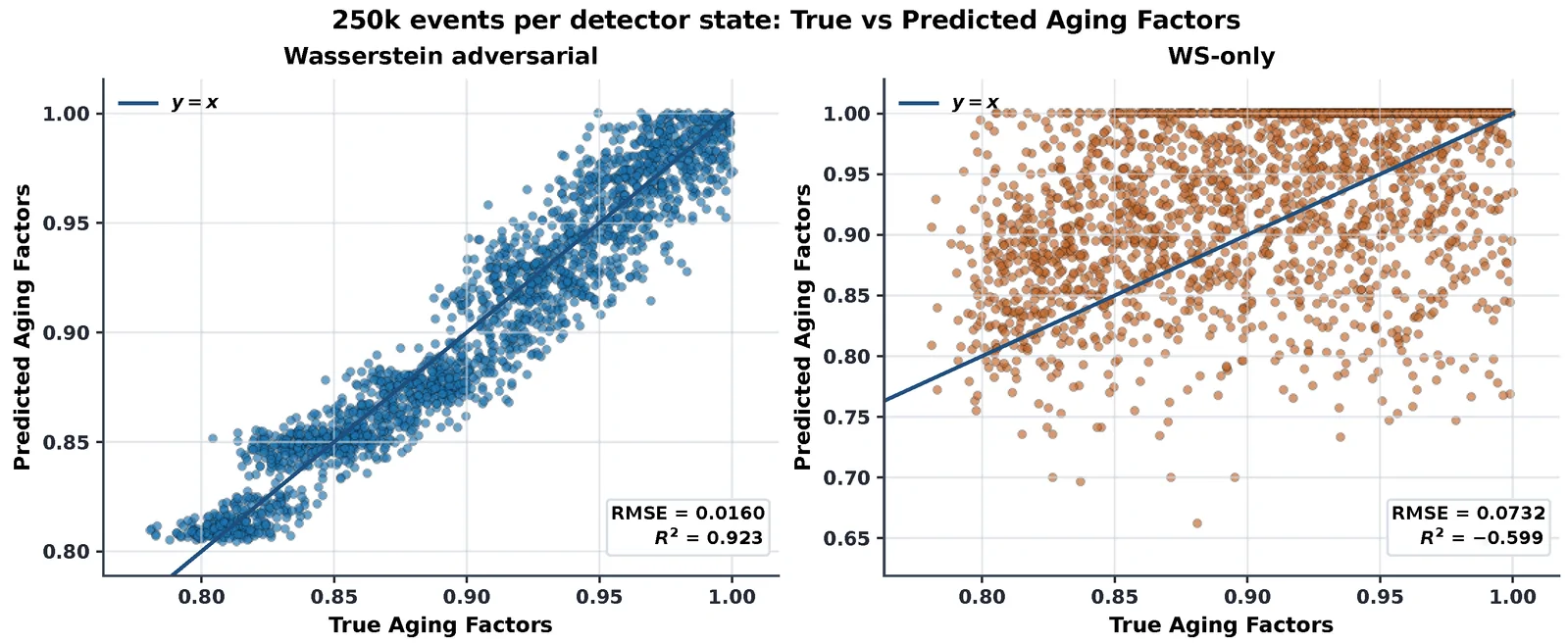
True versus predicted aging coefficients for a representative 250,000 event-per-state run. Ground-truth aging coefficients are used only for evaluation.

Figure 4 shows representative aging-coefficient scatter plots for the 250,000 event setting, using the same active cell list for both methods. The WGAN-style estimates concentrated tightly around the diagonal. The WS-only baseline also improved as the statistics increased, but its finite-sample errors remained larger and its aging-coefficient estimates were obtained independently for each cell.

Aging-coefficient agreement is only one view of calibration quality. The operational goal is to move the measured detector responses back toward the nominal response distribution. In individual cells, this corresponds to reducing the shift between aged and reference energy-reading histograms; at the event level, it corresponds to restoring shower observables such as layer profiles and the total deposited energy. The learned calibration field is therefore interpretable in two complementary ways: it can be compared to injected aging coefficients in simulations, and it can be applied to aged events to inspect whether the corrected response distributions overlap more closely with the reference distributions.

**Figure 5:**
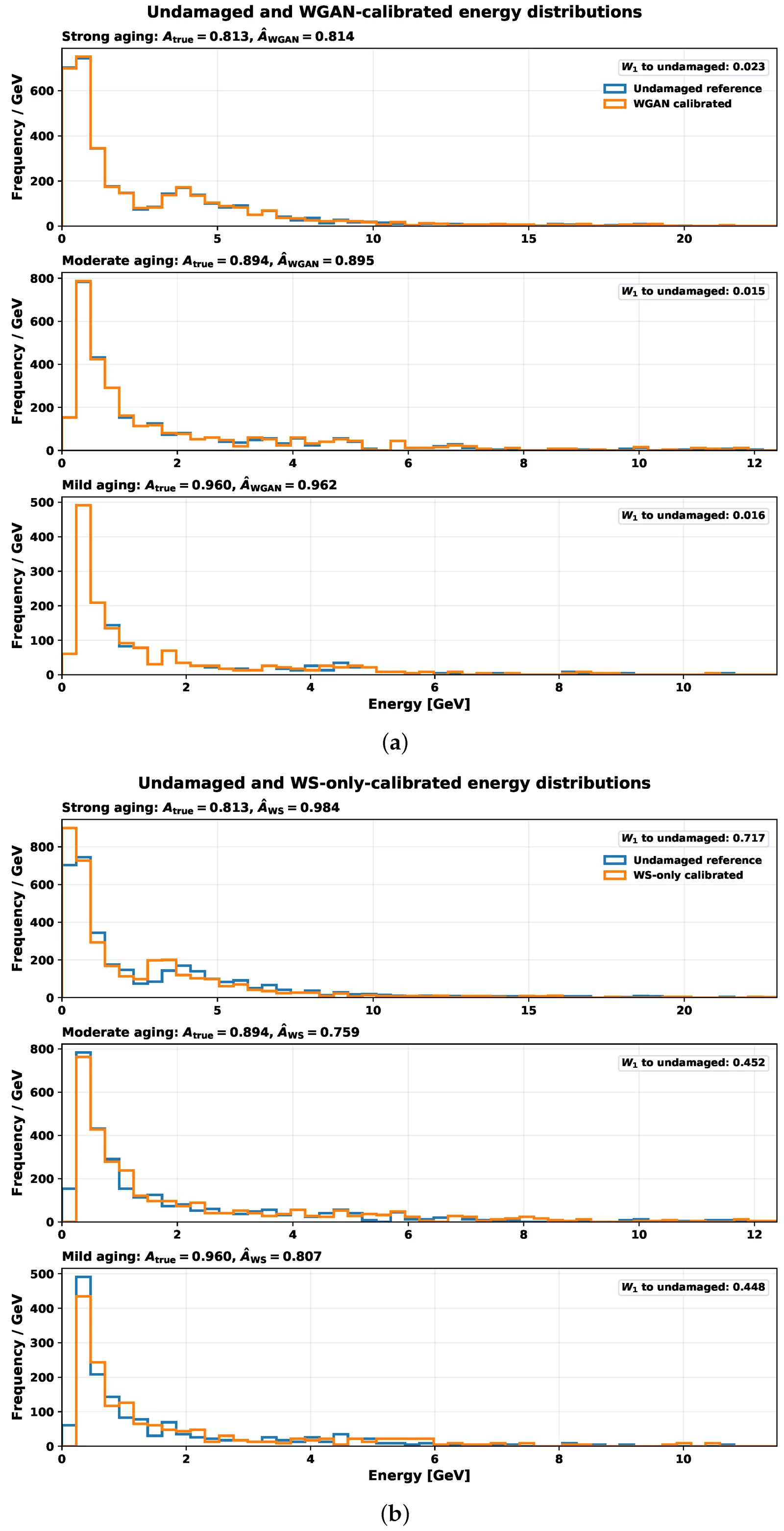
Representative cell-level distributions after applying inverse corrections derived from learned aging coefficients. Reference distributions were compared with calibrated aged responses, so overlap indicates recovery of local sensor response distributions. (**a**) WGAN-calibrated; (**b**) WS-only calibrated.

Figure 5 shows this local response recovery for the same representative cells after applying the inverse corrections derived from the learned aging coefficients. Both methods reduced the pre-calibration mismatch shown in Figure 1, but the WGAN-calibrated distributions provided a tighter agreement with the reference response in the representative examples. This is consistent with the lower aging-coefficient RMSE obtained by the adversarial method, while the WS-only result remained an interpretable marginal-matching baseline.

**Figure 6:**
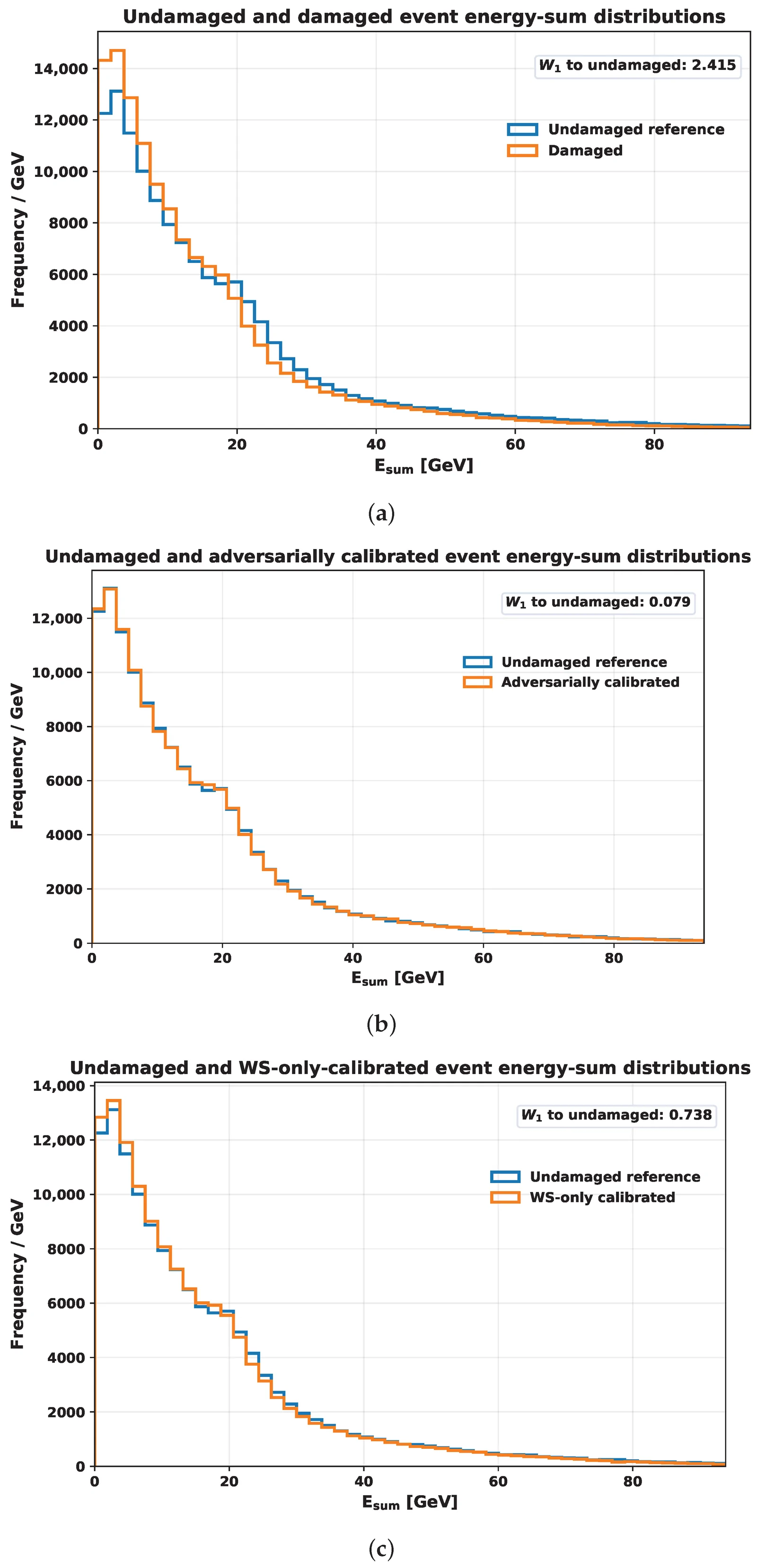
Event-level energy-sum comparisons. The aged response shows the aging-induced energy-scale shift, while applying the learned WGAN and WS-only corrections restores the total deposited-energy distribution toward the reference response. (**a**) Aged baseline; (**b**) WGAN-calibrated; (**c**) WS-only calibrated.

Figure 6 evaluates the calibration at the event level through the total deposited energy. The aged baseline is shifted away from the reference distribution, while both calibrated samples move the energy-sum response back toward the reference. This recovery is a physically meaningful validation beyond cell-level distribution matching: the total event energy tests whether the learned sensor corrections improve a shower-level observable that is directly relevant for calorimeter performance.

## 4. Discussion

Both the mean-energy-ratio and WS-only estimators performed worse than the global-mean predictor in terms of the $R^{2}$; superiority over either baseline alone would not establish a strong advantage. The relevant result is instead that the adversarial field reduced the RMSE below the global-mean reference and achieved a positive $R^{2}$ at every event count. Thus, it recovered part of the cell-wise coefficient variation rather than only a detector-wide average. The two independent estimators remain useful diagnostic controls: the mean-energy ratio tests a direct response-scale estimate, while WS-only tests a distributional cell-wise estimate.

The difference among the tested estimators is not simply the neural network capacity. Both non-adversarial estimators decompose the problem into separate cell-wise marginal estimates. This makes them interpretable, yet it omits constraints from event topology. A calorimeter shower distributes energy coherently across neighboring cells and layers. A candidate calibration field that looks plausible in each isolated cell statistic may still produce an implausible joint event distribution.

From a deployment perspective, the goal is to improve the reliability and longevity of scientific detector systems by reducing the reliance on fully supervised or manually intensive recalibration procedures. The method is intended as a complementary diagnostic and calibration tool, not as a replacement for detector-quality assurance, hardware monitoring, or physics validation. Miscalibration in high-energy physics can bias downstream analyses, so any deployment of data-driven calibration should include uncertainty estimates, independent validation samples, and operational safeguards.

### Identifiability and Local Validation

Distribution matching does not, by itself, guarantee the structural identifiability of the cell-wise calibration field. Distinct coefficient fields may produce similar corrected event distributions, particularly when the coefficient range is narrow and some cells are only sparsely activated. Consequently, agreement in aggregate observables or a low adversarial discrepancy is necessary, but not sufficient evidence of locally correct calibration. In the present simulation, injected coefficients permitted direct cell-level evaluation through the RMSE and $R^{2}$. A positive $R^{2}$ across the repeated fits demonstrated empirical recovery beyond a global-mean predictor; however, other fields may exist that are compatible with the observed distributions.

This limitation is most relevant for low-occupancy cells, for which the response distributions contain comparatively little information about the local coefficient. The occupancy-stratified results in Supplementary Table S2 support this interpretation empirically: high-occupancy cells had a lower adversarial RMSE than low-occupancy cells at both reported event counts. This dependence is evidence about where recovery is better constrained, not proof that the inferred field is unique. Operational deployment would therefore require held-out local-response checks, uncertainty estimates, detector-specific priors, and independent calibration samples. Spatial or layer-wise regularization could further restrict the admissible calibration fields, but such constraints were not evaluated in the present study.

## 5. Conclusions

We present an unsupervised recalibration study for aging high-granularity calorimeter sensor arrays, formulated as Wasserstein distribution matching between a nominal reference response and an aged response. The hierarchical evaluation uses four sampling-seed combinations and three adversarial configured-seed fits per seed combination, while retaining the four seed-combination averages as the statistical units. The expanded benchmark includes an evaluation-only global-mean coefficient predictor, a per-cell mean-energy ratio, and WS-only matching. The adversarial method achieved an RMSE of 0.0238±0.0014 to 0.0173±0.0012 and a positive $R^{2}$ from 0.828±0.022 to 0.910±0.013 across the tested event counts. In every setting, its RMSE was below the 0.0579 global-mean reference, whereas both independent per-cell estimators remained below $R^{2}=0$. An occupancy-stratified evaluation further showed that high-occupancy cells were recovered more accurately than sparsely activated cells. The contribution is therefore evidence of cell-wise coefficient recovery beyond a global mean using full-event structure, not a claim based on a multiplicative improvement over WS-only. The tested event budgets do not establish a universal minimum data requirement, and the present controls do not replace future comparisons with regularized non-adversarial calibration methods or validation using measured detector aging.

## Figures and Tables

**Table 1:** Aging-coefficient benchmark as a function of event count. Values are mean ± sample standard deviation across four seed combinations; for the adversarial method, the three configured-seed fits were averaged within each seed combination first. All methods were evaluated on the same active-cell definition.

Events per State	Global-Mean Predictor ^†^	Mean-Energy Ratio	WS-Only	Wasserstein Adversarial
RMSE				
50k	0.0579	0.1672±0.0013	0.1167±0.0014	0.0238±0.0014
90k	0.0579	0.1424±0.0014	0.0997±0.0010	0.0211±0.0007
150k	0.0579	0.1179±0.0023	0.0861±0.0015	0.0203±0.0005
250k	0.0579	0.0967±0.0014	0.0748±0.0014	0.0173±0.0012
$R^{2}$				
50k	0	−7.353±0.130	−3.067±0.098	0.828±0.022
90k	0	−5.052±0.119	−1.971±0.061	0.866±0.010
150k	0	−3.154±0.164	−1.213±0.075	0.875±0.005
250k	0	−1.793±0.082	−0.673±0.062	0.910±0.013

^†^ The global-mean predictor assigns the mean true aging coefficient over the evaluated cells to every cell. It is an evaluation-only reference defining *R*^2^ = 0 and was not available to any unsupervised calibration methods during training or deployment.

## Data Availability

The simulated data and experiment configuration files used in this study are available from the corresponding author upon reasonable request. The data are not publicly archived due to file-size and storage limitations.

## Supplementary Materials

The following supporting information can be downloaded at: https://www.mdpi.com/article/10.3390/s26165024/s1, Table S1. Individual adversarial Wasserstein fits for every reference/aged sampling-seed combinations and configured seed. The RMSE and $R^{2}$ are the reported cell-level evaluation metrics on the active-cell set. Table S2. Cells are sorted by hit count over all nominal events and divided into three nearly equal groups that were reused for every event count and seed. Values are mean ± sample standard deviation across four seed combinations; adversarial metrics were first averaged over the three configured seeds within each seed combination.

## Abbreviations

The following abbreviations are used in this manuscript:

CNN	Convolutional neural network
HEP	High-energy physics
RMSE	Root-mean-square error
WGAN	Wasserstein generative adversarial network
WS-only	Wasserstein-only
