# Supplementary material for: Data-Efficient Unsupervised Recalibration of Calorimeter Sensor Arrays Using Wasserstein Adversarial Learning
Source: Sensors (Basel). 2026 Aug 7;26(16):5024. doi: 10.3390/s26165024 (PMC13516911; doi:10.3390/s26165024)
Supplement: Supplementary file 1 [file sensors-26-05024-s001.zip › sensors-4416248-supplementary.pdf]

Supplementary Materials for Data-Efficient Unsupervised Recalibration of Calorimeter Sensor Arrays Using Wasserstein Adversarial Learning

Saraa Ali, Vladimir Bocharnikov, Fedor Ratnikov,
Mikhail Hushchyn, Artem Ryzhikov, and Denis Derkach

Table S1: Individual Wasserstein adversarial fits for every nominal reference/aged sampling-seed combination and configured model seed. RMSE and R^2 are cell-level metrics on the common active-cell set. The aging-coefficient field was fixed across all runs. Because an audit of the archived prepared datasets found that some runs with the same nominal sampling-seed combination contained different realized event subsets, these repetitions characterize repeatability of the complete training and data-preparation pipeline under a fixed aging field, rather than optimizer initialization alone.

Nominal sampling seeds	Model seed	50k		90k		150k		250k	
		RMSE	R^2	RMSE	R^2	RMSE	R^2	RMSE	R^2
0–1	42	0.0233	0.838	0.0251	0.812	0.0250	0.813	0.0165	0.919
0–1	43	0.0246	0.819	0.0194	0.887	0.0179	0.904	0.0165	0.919
0–1	44	0.0219	0.856	0.0221	0.854	0.0167	0.917	0.0172	0.912
2–3	42	0.0191	0.891	0.0193	0.889	0.0226	0.847	0.0150	0.933
2–3	43	0.0277	0.771	0.0229	0.843	0.0185	0.897	0.0167	0.916
2–3	44	0.0222	0.853	0.0197	0.884	0.0192	0.890	0.0158	0.925
4–5	42	0.0222	0.852	0.0213	0.864	0.0232	0.839	0.0170	0.913
4–5	43	0.0254	0.808	0.0208	0.871	0.0216	0.861	0.0197	0.884
4–5	44	0.0216	0.861	0.0208	0.870	0.0180	0.903	0.0166	0.918
6–7	42	0.0225	0.848	0.0219	0.857	0.0188	0.895	0.0205	0.874
6–7	43	0.0247	0.818	0.0199	0.882	0.0231	0.841	0.0195	0.886
6–7	44	0.0306	0.720	0.0202	0.878	0.0193	0.889	0.0160	0.923

Table S2: Occupancy-stratified coefficient recovery. Cells were sorted by hit count over the complete nominal sample and divided into three nearly equal groups that were reused for every event count and seed. Values are mean ± sample standard deviation across four nominal sampling-seed combinations; Wasserstein adversarial metrics were first averaged over the three configured model seeds within each combination.

Events per state	Occupancy	Median hits	WS-only RMSE	Wasserstein adversarial RMSE
50k	Low	1,271	0.1282 ± 0.0047	0.0276 ± 0.0027
50k	Medium	2,470	0.1081 ± 0.0018	0.0253 ± 0.0017
50k	High	5,002	0.1127 ± 0.0012	0.0170 ± 0.0005
250k	Low	1,271	0.0710 ± 0.0036	0.0181 ± 0.0010
250k	Medium	2,470	0.0705 ± 0.0009	0.0187 ± 0.0018
250k	High	5,002	0.0824 ± 0.0008	0.0145 ± 0.0008
